# P01-024 – Vascular risk assessment and MMP-3 gene in FMF

**DOI:** 10.1186/1546-0096-11-S1-A28

**Published:** 2013-11-08

**Authors:** S Mir, B Sozeri, K Ozdemir, A Berdeli

**Affiliations:** 1 peadiatric nephrology, Ege University, Izmir, Turkey; 2 pediatric rheumatology, Ege University, Izmir, Turkey; 3 molecular genetic, Ege University, Izmir, Turkey

## Introduction

The patients characterized with chronic subclinical inflammation even during attack-free periods, are now considered to have an increased risk of atherosclerotic complications as well as other autoinflammatory disease. Damage to the arterial wall due to atherosclerosis causes increased arterial stiffness. Pulse wave velocity (PWV), a noninvasive measure of arterial stiffness, is accepted to be an indicator of subclinical atherosclerosis. Cardiovascular disease included various risk markers; blood biomarkers and genetic markers.

Matrix metalloproteinases (MMPs) are closely related proteinases that together are able to degrade all macromolecules of the extracellular matrix. MMPs are potentially implicated in atherogenesis, progression of atherosclerosis. The gene encoding MMP-3 is polymorphic and an insertion (6A)/deletion (5A) polymorphism (5A/6A polymorphism) in the MMP-3 gene may have functional significance in the regulation of its expression. The 5A allele was associated with higher and the 6A allele with lower transcriptional activity. Up to date, the 6A/6A and 5A/6A genotypes were associated with coronary artery disease and carotid atherosclerosis in adults.

## Objectives

We aimed to evaluate the effect of inflammation and the strength of association MMP-3 promoter low- and high-activity genotypes on the increased risk of subclinical atherosclerosis in FMF patients.

## Methods

Forty-seven patients (M/F =21/26) with FMF, and 50 age- and sex-matched controls were recruited. We measured lipid profile (LDL, total cholesterol and lipoprotein a level) and acute phase reactants (APRs) (white blood cells, erythrocyte sedimentation rate, high sensitive C-Reactive Protein and Serum Amyloid A) in attack free period of all patients. Aortic PWV was determined by using an automatic device (Vicorder, Germany) that allowed on-line pulse wave recording and automatic calculation of the PWV. The 5A/6A polymorphism was typed by RFLP-PCR.

## Results

The mean APRs values were not found statistically significant in patients than control. The distribution of the genotypes of the 5A/6A polymorphism in both control and study patients did not differ significantly (40%,32.8% , respectively p>0.05) from those predicted by the Hardy-Weinberg distribution.

The PWV was slightly higher in patients with FMF than in control subjects (P=0.05). Fifteen patients (32%) have PWV values above the average. These patients have also high SAA and lipoprotein-a levels in attack free period. A significant correlation between PWV and lipoprotein a (P<0.001, r=0.67), and SAA level (P<0.001, r=0.52) was found in patients with FMF. There was no detected hypertension. There were no significant differences (p>0.05) in genotype distributions (hyperlipidemia and arterial stiffness index) and allele frequencies between subgroups.

## Conclusion

The results showed that arterial stiffness is correlated with hyperlipidemia and subclinical inflammation in FMF patients. But, the 5A/6A polymorphism of MMP-3 gene may not be linked with appearance and/or progression of arterial stiffness in FMF patients. Our suggestion is that SAA levels as well as the use of therapy monitoring can be predict in cardiovascular disease in patients with FMF.

## Disclosure of interest

None declared.

